
JOURNAL INFORMATION
==============================
NLM Title Abbreviation: South Med Rec
NLM Journal ID: 0027317

The Southern Medical Record

Publisher: Biomedical Journal Digitization Project

ARTICLE INFORMATION
==============================
PMCID: PMC9079543
Article version: 1
Subjects: Editorial and Miscellaneous

Book Notices

Print publication date: 1883 Jul 20
Volume: 13
Issue: 7
First page: 280
Last page: 280
License: This work is free of known copyright restrictions. For more information, please see PMC Back Issue Digitization.
License URL: https://creativecommons.org/publicdomain/mark/1.0/

==============================

### *BOOK NOTICES.*

UNITED STATES DISPENSATORY. Handsomely bound in one volume, Royal 8 vo, Sheep. Price, $8.00. Carefully revised and rewritten, by Horatio C. Wood, M. D., Prof. of Materia Medica and Therapeutics in the University of Pennsylvania; and Joseph P. Remington, Ph. D., Prof. of Theory and Practice of Pharmacy, in the Philadelphia College of Pharmacy, etc., and Samuel Sadtler, F C. S., Ph. D., Prof. of Chemistry in Philadelphia College of Pharmacy and University of Pennsylvania.

This is tbe fifteenth edition of the old popular Dispensatory, well and favorably known to every Doctor and every Druggist in the United States. No other work is so full, complete and satisfactory, and now we have it brought fully up to all the advances in Materia Medica and Pharmacy. Every Physician and every Druggist should have this great work. It is published by J. B. Lippincott, 715 Market street, Philadelphia.

---

### SPECIAL NOTICES.

**Very Handy.**—A full set of Ahl's Splints, containing a splint adapted to every fracture in the body can be bought at A. L. Hernstein's Surgical Instrument Depot in Atlanta at reduced rates, ($25). Address,

A. L. HERNSTEIN, Atlanta, Ga.

**Diabetes.**—The attention of the profession is called to a new remedy for the successful treatment and permanent cure of Diabetes Mellitus, *GILLIFORD'S SOLUTION of ARSENITE of BROMINE.* This remedy has also proved very useful in the treatment of a variety of nervous affections. Manufactured and sold by R. H. GILLIFORD, M. D., Allegheny, Pennsylvania. In ½-pint bottles, $1.00 per bottle, $10.00 per dozen. Sent by express on receipt of price. Sample free to physicians. July, 1883-12 ms.

**Surgical Instruments.**—A branch house of the New York establishment of A. L. HERNSTEIN, has been established in Atlanta, and will constitute a convenient depot whereat anything in the Surgical line can be bought or manufactured. The Profession throughout the South should note this as an important indication of Southern progress, and should show their appreciation of the same by giving this establishment their encouragement and patronage.

McKESSON & ROBBINS.—This great Drug Establishment of New York, has a wide and long established reputation as reliable and eminently successful business men. Their various preparations are of acknowledged excellence and purity, and are unexcelled for the neatness, taste and beauty with which they are presented to the trade. See their advertisement opposite 1st page of reading matter in this Journal.

More of **ELLIOTT'S** SADDLE BAGS are sold than all other patterns combined. One thousand have been shipped to different parts of the country since January 1st. The proprietor invites a thorough investigation and comparison of every Bag in the market. The U. S. Government did this in 1879, and adopted the ELLIOTT. Doctors that do the same thing get the standard article. Send for circular to A. A. MELLIER, 709 Washington Avenue, St. Louis, Mo.

**PEARK, DAVIS & CO.**—This magnificent Drug establishment, located at Detroit, Mich., have, by unremitting perseverance and faithfulness in all their business interests, obtained the confidence and good will of the medical profession throughout the entire country. They have accomplished much for the progress of Medical Science and largely benefitted mankind by the introduction of new and important Drugs. They are entitled to the thanks of the Profession, and justly deserve the high reputation to which they have attained.

**Wm. R. Warner & Co.**—This splendid Drug House, so widely and favorably known, both to the home and foreign trade, continue to maintain their high position. Their preparations are regarded by the profession everywhere as unsurpassed for purity and elegance. In respect to their quinine pills, so deservedly popular, the following certificate has been published:

PHILADELPHIA, PENN., December 22, 1882.

An analysis of seven samples of Quinine Pills, obtained without knowledge of the manufacturers, was made and published in the American Journal of Pharmacy by me, and those made by William R Warner & Co., were found to be correct as to quantity and purity of Quinine. HENRY TRIMBLE, *Analytical Chemist.*

**Purchasing Agency.**—We have established a Purchasing Agency in connection with the RECORD office, by which parties desiring goods of any kind may order through us what they want, which they can obtain at lowest rates and save the expense of a trip to the city. Strict attention to the interest of the purchaser will be observed in the selection of articles. Subscribers to this Journal will be charged no commission for purchases made through this Agency. Cash should accompany every order. Address,

**Dr. R. C. WORD,** Managing Editor, Atlanta.
